# Comparison of the Effects of Lidocaine and Amiodarone on Patients With Cardiac Arrest: A Systematic Review and Meta-Analysis

**DOI:** 10.7759/cureus.56037

**Published:** 2024-03-12

**Authors:** Tanya Sinha, Ibrahim Reyaz, Roba A Ibrahim, Manisha Guntha, Aung K Zin, Grahitha Chapala, Mohan K Ravuri, Sujith K Palleti

**Affiliations:** 1 Medical Education, Tribhuvan University, Kirtipur, NPL; 2 Internal Medicine, Christian Medical College and Hospital, Ludhiana, IND; 3 Internal Medicine, Elrazi University, Khartoum, SDN; 4 Internal Medicine, Sinai-Grace Hospital, Detroit, USA; 5 Internal Medicine, University of Medicine, Mandalay, Mandalay, MMR; 6 Medicine and Surgery, Mkhitar Gosh Armenian Russian International University, Yerevan, ARM; 7 Nephrology, Louisiana State University Health Sciences Center, Shreveport, USA

**Keywords:** meta-analysis, mortality, cardiac arrest, lidocaine, amiodarone

## Abstract

The objective of this study was to compare the impact of amiodarone and lidocaine on survival and neurological outcomes following cardiac arrest. A systematic review of randomized controlled trials (RCTs) as well as cohort and cross-sectional trials was undertaken, adhering to the Preferred Reporting Items for Systematic Reviews and Meta-Analyses (PRISMA) guidelines. Potential relevant studies were searched in databases, including PubMed, Embase, Cochrane Library, and Web of Science, from the beginning of databases to February 15, 2024. Outcomes assessed in this study were survival to hospital discharge, survival to hospital admission or 24 hours, favorable neurological outcomes, and return of spontaneous circulation (ROSC). A total of seven studies (five observational and two RCTs) were included in this meta-analysis encompassing 19,081 patients with cardiac arrest. Pooled analysis showed no difference between amiodarone and lidocaine in terms of survival to hospital discharge (odds ratio (OR): 0.88, 95% confidence interval (CI): 0.75 to 1.04), ROSC (OR: 0.94, 95% CI: 0.84 to 1.05, p-value: 0.25), favorable neurological outcomes (OR: 0.88, 95% CI: 0.66 to 1.17, p-value: 0.38), and survival to 24 hours (OR: 0.82, 95% CI: 0.55 to 1.21, p-value: 0.31). While lidocaine demonstrated a slight survival advantage, the differences were statistically insignificant. Similarly, no significant variations were observed in ROSC incidence, neurological outcomes, or survival at 24 hours. These findings align with current guidelines but underscore the necessity for further rigorous RCTs to provide conclusive recommendations.

## Introduction and background

In the United States, in 2019, there were an estimated 232,000 visits to the emergency department (ED) due to cardiac arrest. The incidence rate of cardiac arrest was roughly 0.2% [1]. Roughly 12% of out-of-hospital cardiac arrest (OHCA) patients managed to survive until their discharge from the hospital [2], and among these survivors, around 50% exhibit favorable neurological outcomes [3]. Nevertheless, some individuals who survive cardiac arrest experience subtle cognitive challenges [4], thereby presenting cardiac arrest as a significant public health issue and the primary cause of mortality globally, thereby imposing substantial burdens on patients and society [5].

Recent guidelines from the International Liaison Committee on Resuscitation regarding resuscitation recommend amiodarone and lidocaine as the preferred treatments for OHCA patients experiencing ventricular fibrillation (VF)/pulseless ventricular tachycardia (pVT) [6]. Amiodarone prolongs the duration of the third phase of heart conduction cell action potentials, reducing potassium ion outflow from these cells [7]. Conversely, lidocaine inhibits impulse conduction in nerve fibers reversibly by blocking the potassium sodium pump and hindering neuron membrane permeability to sodium ions [8]. Khan et al. conducted a systematic review and network meta-analysis comparing amiodarone, lidocaine, magnesium, and placebo for OHCA patients with VF/pVT, indicating that lidocaine may be the most effective anti-arrhythmic drug for increasing survival to hospital discharge in patients with pulseless VF/pVT [9]. Before the 2000 guidelines for cardiopulmonary resuscitation, lidocaine was the preferred antiarrhythmic drug for patients with shock-resistant VF [10]. However, comparative studies with amiodarone altered this recommendation, with lidocaine now being suggested only when amiodarone is unavailable [11]. Nevertheless, ongoing debates persist regarding the choice of antiarrhythmic drugs in cases of defibrillation-resistant VF and pVT.

The objective of this study was to systematically evaluate the existing literature and conduct a meta-analysis to ascertain the impact of amiodarone and lidocaine on survival and neurological outcomes following shock-refractory cardiac arrest.

## Review

Methodology

A systematic review of randomized controlled trials (RCTs) as well as cohort and cross-sectional trials was undertaken, adhering to the Preferred Reporting Items for Systematic Reviews and Meta-Analyses (PRISMA) guidelines [12]. Potential relevant studies were searched in databases, including PubMed, Embase, Cochrane Library, and Web of Science, from their inception to February 15, 2024. A structured search was conducted using the search terms "cardiac arrest," "lidocaine," and "amiodarone," in combination with Medical Subject Heading (MeSH) terms and Boolean algebra operators (AND, OR). No language restrictions were applied to the retrieved articles. Additionally, manual searches of reference lists, related citations, and gray literature from websites were performed. Two authors conducted the search, with any discrepancies resolved through discussion.

Study Selection

Studies were deemed eligible for inclusion in the meta-analysis if they met the PICOS (population, intervention, comparator, outcome, and study design) criteria. The population consisted of cardiac arrest patients. The intervention involved intravenous amiodarone, while the control group received lidocaine. Outcome measures included survival to hospital discharge, survival to hospital admission or 24 hours, favorable neurological outcome, and return of spontaneous circulation (ROSC). Accepted study designs included RCTs and retrospective studies, while review articles, case reports, case series, reviews, and editorials were excluded. All relevant studies were imported into EndNote X9 (Clarivate, London, UK), where duplicate literature was eliminated. Two researchers independently screened studies based on titles and abstracts, with any disagreements resolved through discussion. Irrelevant studies that did not meet the PICOS criteria were excluded. In cases of uncertainty, a third author was consulted.

Data Extraction and Quality Assessment

Data from the included studies were independently extracted by two reviewers using a pre-designed Microsoft Excel sheet (Microsoft Corporation, Redmond, WA). Information extracted included author details, study design, publication year, sample size, intervention, and control groups. Outcomes assessed in this study were survival to hospital discharge, survival to hospital admission or 24 hours, favorable neurological outcome, and ROSC. Quality assessment was performed using the Cochrane Risk of Bias assessment tool for RCTs and the Newcastle-Ottawa Scale for observational studies.

Statistical Analysis

The Mantel-Haenszel method was employed for analyzing dichotomous outcomes. Results are presented as odds ratios (OR) with corresponding 95% confidence intervals (CI) and two-tailed p-values. A p-value less than 0.05 was considered statistically significant. Statistical heterogeneity was assessed using the X^2^ (Cochran Q) test. Heterogeneity was deemed present if Q > df (degrees of freedom) and confirmed if p ≤ 0.10. The extent of heterogeneity was quantified using I-square values, categorized as none (0-24.9%), low (25-49.9%), moderate (50-74.9%), and high (>75%). The random-effects model was applied irrespective of the heterogeneity among the study results to deal with the possible variation due to population characteristics, sample size, and study design. Statistical analysis was performed using RevMan version 5.4.1 (The Cochrane Collaboration, London, UK).

Results

**Figure 1:**
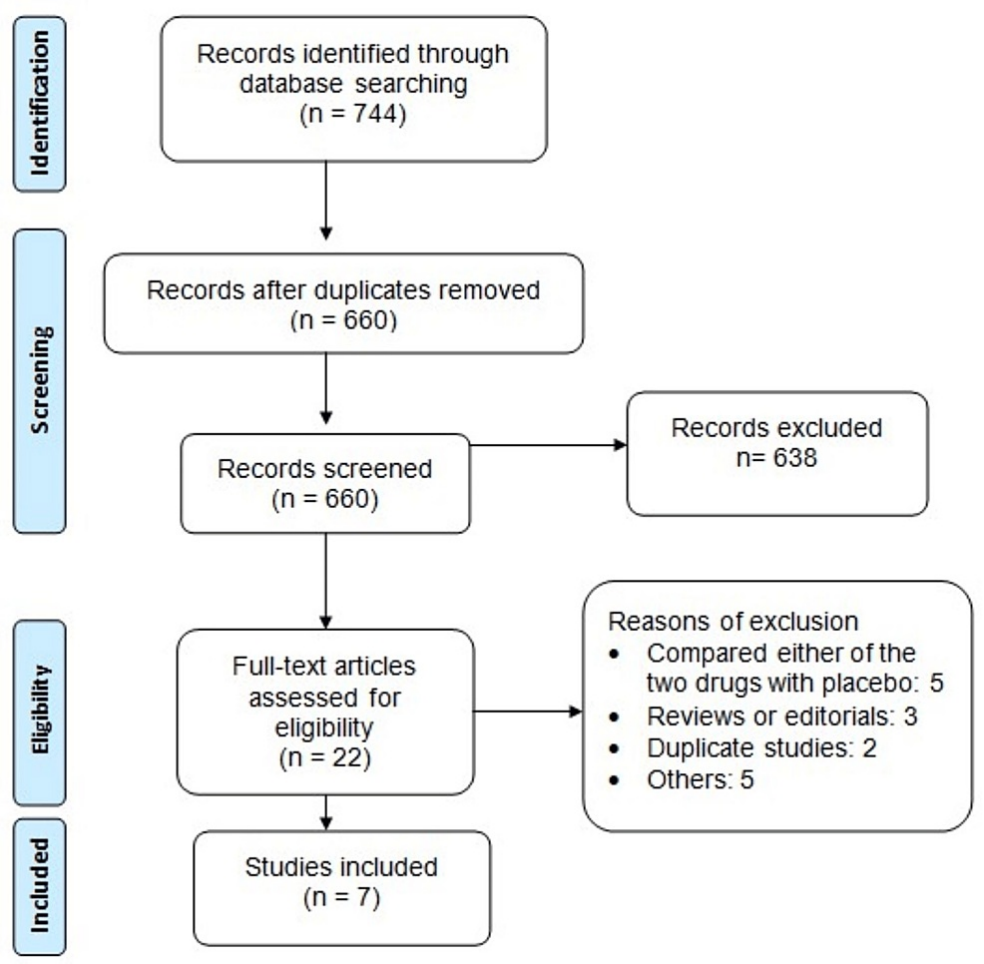
PRISMA flowchart of study selection PRISMA: Preferred Reporting Items for Systematic Reviews and Meta-Analyses.

Figure 1 depicts the methodology employed for trial screening and selection. The initial search strategy identified 744 pertinent articles, out of which 84 were identified as duplicates. Following the exclusion of duplicated entries, a total of 660 studies were initially reviewed using abstracts and titles. The full text of 22 articles underwent further scrutiny. Ultimately, seven articles met the inclusion criteria for this meta-analysis. The studies encompassed in this meta-analysis spanned publication dates from 2002 to 2022. Among them, two studies were RCTs, while the remainder comprised observational studies. Table 1 shows the characteristics of the included studies. The pooled sample size was 19,081. In all included studies, the majority of participants were males. The number of males enrolled in included studies ranged from 91 to 9346. Table 2 presents the quality assessment of the included studies.

**Table 1:** Characteristics of included studies RCT: randomized controlled trial.

Author	Year	Study design	Groups	Sample size	Mean age (years)	Males (n)
Dorian et al. [13]	2002	RCT	Amiodarone	180	68	136
			Lidocaine	167	68	136
Kishihara et al. [14]	2022	Observational	Amiodarone	189	69	143
			Lidocaine	63	68	49
Kudenchuk et al. [15]	2016	RCT	Amiodarone	974	63.7	762
			Lidocaine	993	63	816
Rea et al. [16]	2006	Observational	Amiodarone	79	62	49
			Lidocaine	74	64	61
Tagami et al. [17]	2016	Observational	Amiodarone	801	66.9	600
			Lidocaine	801	67.3	616
Wagner et al. [18]	2022	Observational	Amiodarone	10,058	65.2	6478
			Lidocaine	4572	65.7	2868
Wang et al. [19]	2020	Observational	Amiodarone	113	68.2	76
			Lidocaine	17	59.1	15

**Table 2:** Quality assessment of included studies

Risk of bias assessment of observational studies							
Study ID	Selection	Comparison	Outcome and exposure assessment	Overall			
Tagami et al. [17]	3	2	3	Good			
Wang et al. [19]	4	2	2	Good			
Kishihara et al. [14]	3	1	2	Fair			
Rea et al. [16]	3	2	3	Good			
Wagner et al. [18]	3	2	3	Good			
Risk of bias assessment of randomized control trials							
Study ID	Randomization	Allocation concealment	Blinding of personnel and participants	Blinding of outcome assessment	Incomplete outcome data	Selective reporting	Other bias
Dorian et al. [13]	No bias	No bias	No bias	No bias	No bias	No bias	No bias
Kudenchuk et al. [15]	No bias	No bias	No bias	No bias	No bias	No bias	No bias

Meta-Analysis of Outcomes

**Figure 2:**
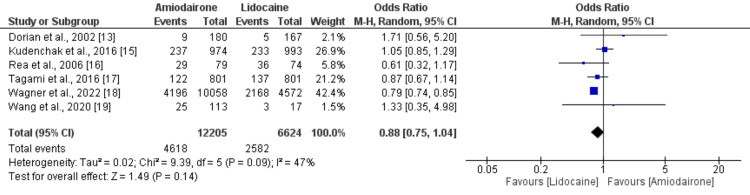
Forest plot comparing survival to hospital discharge between two groups Sources: [13,15-19].

Survival to hospital discharge: Six studies provided data on survival to hospital discharge. The pooled analysis revealed that the rate of survival to hospital discharge was slightly higher among patients administered lidocaine (38.98%) in comparison to those receiving amiodarone (37.83%). However, this difference did not reach statistical significance (OR: 0.88, 95% CI: 0.75 to 1.04), as illustrated in Figure 2. Low heterogeneity was observed among the study outcomes (I-square: 47%).

**Figure 3:**
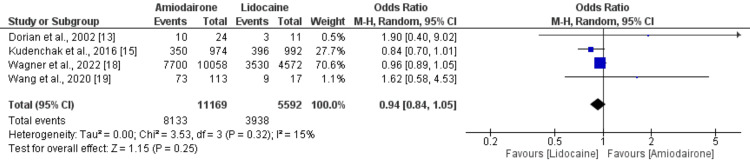
Forest plot comparing the return of spontaneous circulation (ROSC) between two groups Sources: [13,15,18,19].

Return of spontaneous circulation: Forest plots illustrating the impact of management on the occurrence of ROSC are depicted in Figure 3. A total of four studies were encompassed in the pooled analysis of ROSC. The pooled analysis indicated no significant difference in ROSC rates (OR: 0.94, 95% CI: 0.84 to 1.05, p-value: 0.25). Moreover, no heterogeneity was observed among the study outcomes (I-square: 15%).

**Figure 4:**
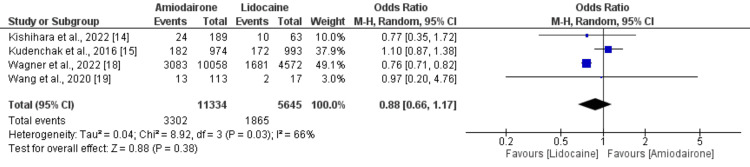
Forest plot comparing favorable neurological outcomes between study groups Sources: [14,15,18,19].

Favorable neurological outcomes: Four studies compared favorable neurological outcomes between lidocaine and amiodarone groups and the results are shown in Figure 4. Pooled analysis of four studies did not report any significant differences in terms of favorable neurological outcomes between amiodarone and lidocaine groups (29.13% vs. 33.04%) (OR: 0.88, 95% CI: 0.66 to 1.17, p-value: 0.38). Moderate heterogeneity was reported among the study results (I-square: 66%).

**Figure 5:**
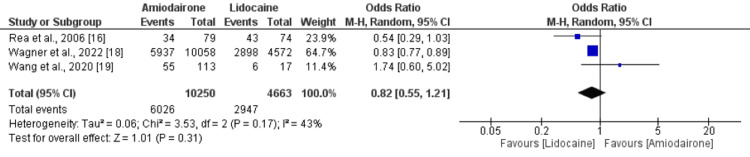
Forest plot comparing survival to 24 hours between study groups Sources: [16,18,19].

Survival to 24 hours: Three studies reported survival to 24 hours and the results are shown in Figure 5. The pooled analysis reported no significant difference between the two groups in terms of survival to 24 hours (OR: 0.82, 95% CI: 0.55 to 1.21, p-value: 0.31). Low heterogeneity was reported among the study results (I-square: 43%).

Discussion

The meta-analysis incorporated RCTs and observational studies investigating the utilization of amiodarone and lidocaine in patients experiencing sudden cardiac arrest. We evaluated studies comparing amiodarone and lidocaine concerning their impact on various parameters related to the ROSC, survival to hospital admission, survival to 24 hours, and discharge from the hospital with a favorable neurological outcome. Notably, our analysis revealed no significant difference between amiodarone and lidocaine across any of the outcomes assessed in this meta-analysis.

Both lidocaine and amiodarone have been widely employed for managing ventricular arrhythmias, including shockable rhythms such as VF and ventricular tachycardia (VT) [20]. In cases where shockable rhythms persist despite attempts at defibrillation, both medications are considered viable treatment options [21]. Current guidelines recommend administering these drugs after the third defibrillation attempt, applicable to both adults and children. This meta-analysis evaluates the effectiveness of pharmacological treatments (amiodarone vs. lidocaine) in cases of sudden cardiac arrest. The ROSC serves as a fundamental metric for assessing resuscitation efficacy [22]. Our pooled analysis found no significant statistical differences between the groups treated with amiodarone or lidocaine in terms of ROSC. This parameter holds critical importance for resuscitation efforts in both non-hospital and in-hospital settings [23].

A crucial determinant of treatment outcome is survival to hospital discharge. While there is no significant difference between the two drugs regarding survival to hospital discharge, patients receiving lidocaine exhibited higher survival rates. Similar findings were echoed in the network meta-analysis conducted by Wang et al. [6], which indicated that lidocaine attained a superior recommendation level (surface under the cumulative ranking (SUCRA) rank) compared to all other medications in terms of survival to hospital admission at various follow-up durations. Additionally, Sanfilippo et al. [24] conducted a pairwise meta-analysis and concluded that there was no statistically significant difference between amiodarone and lidocaine in terms of long-term survival to hospital admission. Considering the number of studies included, further relevant research is warranted in the future to establish a more robust conclusion.

In two trials, amiodarone was found to improve survival to admission compared to placebo, although no significant difference was observed in survival to hospital discharge or neurologically intact survival. It is worth noting that both trials were underpowered to detect these outcomes [13,25]. Additionally, a recent study demonstrated that a shorter duration from the 9-1-1 call to the administration of amiodarone was associated with a higher likelihood of ROSC upon arrival at the emergency department compared to placebo [23]. This study also indicated that delayed administration of amiodarone was linked to reduced probabilities of ROSC compared to placebo [23].

The findings of this meta-analysis are in accordance with current guidelines established by the American Heart Association (AHA) and the European Resuscitation Council (ERC). These guidelines suggest considering amiodarone or lidocaine as Class IIb recommendations. Specifically, administering a 300 mg bolus of amiodarone after three defibrillation attempts, or using a 1-1.5 mg/kg bolus of lidocaine as an alternative if amiodarone is not available [26,27]. The 2015 ERC and AHA guidelines were formulated based on available data, with ongoing randomized clinical trials exploring the comparative use of amiodarone, lidocaine, and placebo. The pooled data from this meta-analysis indicate that lidocaine's use should not be restricted to situations where amiodarone is unavailable, as both drugs demonstrate similar efficacy. Amiodarone acts as a membrane-stabilizing antiarrhythmic drug, improving the response to defibrillation in VF or hemodynamically unstable VT [28]. Although its onset of antifibrillatory action is slower compared to lidocaine, its effects are more sustained [29]. Lidocaine has historically been employed as one of the antiarrhythmic drugs for shock-resistant or recurrent VF [30]. Notably, lidocaine may compromise counter-shock efficacy due to increased affinity for sodium channel receptors in acidic environments [31]. An RCT by Kudenchuk et al. [15] demonstrated substantial equivalence in short-term outcomes between amiodarone and lidocaine. However, the amiodarone group exhibited more frequent episodes of hypotension and bradycardia, despite the utilization of a new amiodarone aqueous formulation devoid of hypotensive effects, which replaced polysorbate 80 with Captisol. Consequently, although current guidelines advocate for amiodarone as the primary choice in refractory and recurrent VF/pVT cases, recent evidence may prompt discussions and revisions regarding the consensus on scientific recommendations [32]. Further clinical trials with larger sample sizes are imperative to corroborate these findings.

Study Limitations

While our systematic review and network meta-analysis offer valuable insights, several limitations should be acknowledged. Firstly, our meta-analysis comprised only seven studies, indicating a relatively small sample size. Including more studies would enhance the statistical robustness of our analysis. Additionally, all included studies lacked long-term follow-up data, highlighting the necessity for future investigations with extended follow-up periods. Despite these limitations, our study represents the most recent meta-analysis assessing the efficacy and safety of anti-arrhythmic drugs in patients experiencing cardiac arrest. Nonetheless, further verification through a larger number of RCTs is warranted.

## Conclusions

In summary, our meta-analysis of seven studies encompass RCTs and observational studies. While lidocaine demonstrated a slight survival advantage, the differences were statistically insignificant. Similarly, no significant variations were observed in ROSC incidence, neurological outcomes, or survival at 24 hours. These findings align with current guidelines but underscore the necessity for further rigorous RCTs to provide conclusive recommendations. Despite amiodarone's historical preference, recent evidence suggests comparable efficacy with lidocaine, necessitating a reevaluation of treatment strategies. Future clinical trials with larger sample sizes are imperative for confirming these results and guiding clinical practice.
